# Respiratory mucosal immunity: Biological functions, diseases, prevention and therapy

**DOI:** 10.1016/j.gendis.2026.102200

**Published:** 2026-04-21

**Authors:** Yu Zhang, Zhiruo Song, Weiqi Hong, Xuemei He, Xiawei Wei

**Affiliations:** Laboratory of Aging Research and Cancer Drug Target, State Key Laboratory of Biotherapy and Cancer Center, National Clinical Research Center for Geriatrics, West China Hospital, Sichuan University, Chengdu, Sichuan 610041, China

**Keywords:** Respiratory infections, Respiratory mucosal immunity, Respiratory mucosal vaccines, Vaccine delivery and immune enhancement strategies, Vaccine development

## Abstract

Respiratory mucosal (RM) immunity is a highly specialized and dynamic network that safeguards the airways from inhaled pathogens while preserving tissue homeostasis. Acting as the body’s first line of defense, RM immunity integrates immune tolerance, barrier protection, immune surveillance, tissue repair, and the establishment of long-term immunological memory. Dysregulation of these processes contributes to a broad spectrum of diseases, including acute viral and bacterial infections, fungal colonization, and chronic inflammatory disorders, highlighting the urgent need for effective preventive strategies targeting the respiratory mucosa. The unprecedented global impact of coronavirus disease 2019 (COVID-19) has further highlighted this need and catalyzed rapid advances in vaccines capable of inducing both local and systemic immunity at the respiratory portal of entry, alongside progress in inhalable antibody therapies. This review first summarizes the principal biological functions of the respiratory mucosa and the underlying mechanisms, followed by an overview of immune dysregulation associated with respiratory diseases. It then highlights recent advances in mucosal intervention strategies, with a particular focus on the development of RM vaccine platforms—including live-attenuated, inactivated, viral vector, protein subunit, and mRNA vaccines. It further discusses next-generation RM vaccine strategies emphasizing upper airway immunity, broadened antigen design and intranasal safety. Together, these advances provide a conceptual and translational framework for advancing RM-based interventions against respiratory pathogens.

## Introduction

Mucosal tissues are continuously exposed to the external environment, constituting the body’s first line of defense against external invasion. Among them, the respiratory mucosa plays a particularly critical role, as it is constantly exposed to inhaled allergens, pollutants, and pathogens such as bacteria and viruses during normal respiration.^1^ Pathogens frequently exploit these mucosal surfaces as their initial portals for infection.^2^ For this reason, the respiratory mucosa possesses robust mechanical and chemical defense mechanisms to degrade and eliminate most foreign substances.^3^ Moreover, the respiratory mucosa is equipped with a complex, highly specialized immune system integrating both innate and adaptive immune components, providing comprehensive protection against potential pathogenic threats while maintaining tissue integrity and immune homeostasis.^4^^,^^5^

The anatomy of the respiratory tract further emphasizes its susceptibility. The upper respiratory tract (URT), comprising the nasal cavity, pharynx, and larynx, is the primary entry site for numerous pathogens, including influenza viruses and severe acute respiratory syndrome coronavirus 2 (SARS-CoV-2). Early infection in the URT often precedes dissemination to the lower respiratory tract (LRT), which includes the trachea, bronchi, and bronchioles, where disease can become severe. This cascade highlights the importance of establishing robust mucosal immunity at the portals of entry to prevent pathogen colonization and protect the lower airways from damage.^6^^,^^7^ Beyond serving as a physical interface, the respiratory mucosa performs multiple coordinated biological functions that are essential for maintaining pulmonary integrity and host survival. These include immune tolerance, barrier defense, immune surveillance, tissue repair, and immunological memory.^8^, ^9^, ^10^, ^11^, ^12^ Together, these functions ensure that immune activation is tightly balanced with tolerance, enabling rapid pathogen elimination without excessive inflammation or tissue damage. Dysregulation of these functions is implicated in the onset and progression of various respiratory diseases, including acute viral and bacterial infections, fungal colonization, and chronic inflammatory conditions such as asthma and chronic obstructive pulmonary disease (COPD).^4^^,^^13^^,^^14^ The breakdown of mucosal immune homeostasis not only predisposes individuals to recurrent infections but also drives persistent inflammation and airway remodeling, thereby linking mucosal immunity to both infectious and noninfectious respiratory pathologies.^13^^,^^14^

The COVID-19 pandemic has notably accelerated our understanding of respiratory mucosal (RM) immunity, underscoring the urgent need to develop preventive and therapeutic strategies capable of inducing both local and systemic immune responses at the respiratory portal of entry 15, 16, 17. This global crisis has not only redefined the central role of the mucosal barrier in host defense against infection but also catalyzed unprecedented innovation in mucosal immunization technologies. Among these, RM vaccines have emerged as one of the most promising approaches, capable of eliciting localized protective immunity and achieving rapid sterilizing effects at the site of infection, while simultaneously inducing systemic immune responses.^18^ In parallel, the advance of inhalable antibody therapies has further expanded respiratory mucosa-based interventions, offering new avenues for rapid protection and early therapeutic intervention.^19^ Robust mucosal immune responses involve interferons (IFNs), secretory immunoglobulin A (SIgA), mucosal plasma cells, and tissue-resident memory T cells (TRM cells), alongside T helper 17 cell (Th17)-mediated responses.^20^, ^21^, ^22^, ^23^ These coordinated mechanisms enhance local viral clearance, prevent downward pathogen dissemination, and promote the establishment of systemic immunological memory. Consequently, vaccines occupy a central position in RM immunity—they not only serve as key drivers of localized defense but also bridge innate and adaptive immunity to achieve long-lasting protection.^23^^,^^24^ However, the complex anatomical structure and physiological barriers of the respiratory mucosa pose significant challenges for vaccine delivery.^24^ Although numerous mucosal vaccine candidates have demonstrated immunogenicity in preclinical models, translation to licensed human vaccines has been slow, reflecting challenges in delivery, durability, and regulation.^25^^,^^26^

This review provides a comprehensive overview of RM immunity, spanning its biological functions, relevance to respiratory diseases, and clinical translational applications. We first summarize the biological functions and regulatory mechanisms of RM, with emphasis on barrier protection, immune surveillance, and the establishment of long-term immunological memory. We then discuss diseases associated with dysregulated RM immunity, including viral, bacterial, and fungal infections as well as chronic inflammatory airway disorders, highlighting their underlying immunopathological features. Building on these foundations, we examine prevention and therapeutic strategies based on RM immunity, with particular emphasis on inhalable antibody approaches and RM vaccine platforms, including live-attenuated, inactivated, protein subunit, viral vector and mRNA vaccines. We further propose three guiding principles for next-generation RM vaccine development: prioritizing URT—localized immunity and precise mucosal immune programming; advancing antigen design from strain-matched approaches toward broad-spectrum and preparedness-oriented strategies; and ensuring the safety of intranasal vaccines for durable, population-wide application. Finally, we analyze key translational barriers limiting clinical implementation and discuss potential solutions. Collectively, this review aims to provide integrated insights and forward-looking perspectives to inform the rational design of next-generation mucosal vaccines against respiratory pathogens.

## Defense mechanisms and biological functions of the respiratory mucosa

The RM immune system consists of two functional compartments: inductive sites that initiate adaptive immune responses and effector sites where immune reactions are executed.^24^ The inductive sites include nasal-associated lymphoid tissue (NALT) and bronchus-associated lymphoid tissue (BALT).^2^^,^^27^ In humans, NALT comprises the pharyngeal, lingual, palatine, and tubal tonsils, collectively forming Waldeyer’s ring, which serves as the primary immune sentinel of the respiratory mucosa.^28^ BALT, typically located at bronchial bifurcations, contains organized lymphoid follicles that initiate immune responses against pathogens entering the LRT.^27^^,^^29^^,^^30^ Lymphocytes activated at these sites subsequently migrate to effector tissues such as the lamina propria and glandular stroma through integrin- and chemokine-mediated homing, linking antigen recognition to local immune defense.^24^

Within this network, the RM immune system maintains respiratory health through three coordinated defense layers: epithelial barriers, innate immunity, and adaptive immunity.^31^^,^^32^ Together, they support five key biological functions: immune tolerance and homeostasis, epithelial barrier protection, pathogen clearance, tissue repair, and long-term immune memory. Immune tolerance toward commensal microbes and harmless inhaled antigens is largely maintained by regulatory T cells (Tregs) and anti-inflammatory cytokines such as interleukin-10 (IL-10) and transforming growth factor-β (TGF-β). The epithelial barrier prevents microbial invasion through tight junctions, mucus-ciliary clearance, antimicrobial peptides (AMPs), and SIgA. When these barriers are breached, innate and adaptive immune responses cooperate to eliminate invading pathogens. Following pathogen clearance, tissue repair mechanisms restore epithelial integrity, while TRM cells, memory B cells (MBCs), and long-lived plasma cells (LLPCs) establish durable immune protection.^8^, ^9^, ^10^, ^11^, ^12^ In this section, we highlight recent advances in mucosal LLPCs and MBCs—a dimension previously underexplored yet fundamental for durable humoral immunity. The functional synergy of LLPCs and MBCs represents an emerging paradigm for long-term mucosal antibody memory, an insight poised to reshape vaccine strategies for sustained respiratory protection.^33^^,^^34^

## Immune tolerance and homeostasis

Immune tolerance in the respiratory tract enables coexistence with commensal microbiota and harmless inhaled antigens while preserving responsiveness to pathogens. Tregs, a subset of CD4^+^ T cells with potent immunosuppressive functions, are central to maintaining immune homeostasis and self-tolerance.^35^^,^^36^ Their differentiation and activity depend on the transcription factor Forkhead box P3 (Foxp3), and loss-of-function mutations in Foxp3 lead to severe immune dysregulation characterized by autoimmunity and allergic disease.^37^^,^^38^ Tregs restrain excessive immune activation through several complementary mechanisms that preserve tissue integrity while preventing inflammation. These include secretion of anti-inflammatory mediators such as IL-10, TGF-β, IL-35, and fibrinogen-like protein 2, which suppress effector T-cell activity and pro-inflammatory cytokine production; ectoenzymes including CD39 and CD73, which convert pro-inflammatory extracellular adenosine triphosphate into immunosuppressive adenosine; and inhibitory receptors such as CTLA-4, PD-1, LAG-3, and Galectin-1 that mediate contact-dependent suppression by limiting costimulatory signaling in antigen-presenting cells (APCs) or delivering inhibitory signals to effector T cells.^35^^,^^36^

At the mucosal interface, dendritic cells (DCs) can acquire tolerogenic phenotypes in response to microbial metabolites, including short-chain fatty acids and vitamin A derivatives. These tolerogenic DCs promote the differentiation of naïve T cells into Foxp3^+^ Tregs through the secretion of TGF-β and retinoic acid.^39^^,^^40^ In parallel, metabolites derived from respiratory epithelial cells and commensal microbiota further reinforce Treg differentiation and expansion, stabilizing a local immunoregulatory microenvironment.^41^

Innate lymphoid cells (ILCs) also contribute to maintaining mucosal equilibrium.^42^ Predominantly located in barrier tissues, ILCs are classified into three major subsets—ILC1, ILC2, and ILC3—based on transcriptional profiles. Under steady-state conditions, ILC2 and ILC3 support epithelial integrity and mucosal homeostasis through cytokines such as IL-5, IL-13, and IL-22 that promote tissue repair and limit inflammation. During infection or barrier disruption, ILC1 and ILC3 produce IFN-γ and IL-17/IL-22, respectively, enhancing antimicrobial defense and coordinating adaptive immune recruitment. Through these coordinated functions, ILC subsets help maintain the balance between immune tolerance and immune surveillance in the respiratory mucosa.^5^^,^^42^

## Epithelial barrier defense and mucosal protection

The epithelial barrier constitutes the first line of RM immunity. It employs a multilayered strategy that integrates physical, mechanical, and chemical defenses to neutralize most invaders before immune activation.^43^

When pathogens enter the respiratory tract, epithelial cells are tightly connected by intercellular junctions that prevent bacteria, viruses, and particulate matter from penetrating the mucosal surface.^8^ This barrier is dynamically regulated rather than static. Studies show that IL-22 upregulates tight-junction proteins, strengthening epithelial cohesion and preserving mucosal integrity.^44^ The airway surface is coated with mucus secreted by goblet cells, which traps inhaled microbes and particles. The coordinated beating of cilia then moves the mucus toward the pharynx, clearing trapped pathogens through the mucociliary clearance system.^45^, ^46^, ^47^ Within the mucus layer, SIgA produced by subepithelial plasma cells neutralizes pathogens, thereby preventing their attachment and invasion. In addition, respiratory epithelial cells sense environmental stimuli and release AMPs, such as β-defensins and LL-37/hCAP-18, into the mucus layer.^48^ These peptides kill pathogens mainly by disrupting bacterial membranes or interfering with essential intracellular processes after entry.^48^, ^49^, ^50^ Together, these mechanisms maintain airway microbial balance and protect respiratory health.

## Innate and adaptive immune surveillance and response

Immune surveillance and pathogen clearance are central functions of the RM immune system, coordinated by innate and adaptive immunity. When epithelial barriers are compromised, the respiratory mucosa rapidly initiates immune responses to control invading pathogens.

## Innate immunity in the respiratory mucosa

Once pathogens breach the epithelial barrier, the innate immune system is rapidly mobilized to eliminate invaders and alert adaptive immunity. Macrophages, neutrophils, and IFNs form the core of this early defense response.^9^^,^^51^

Macrophages exhibit functional plasticity during infection.^10^^,^^52^ In the early inflammatory phase, macrophages adopt a pro-inflammatory phenotype that produces cytokines such as TNF-α, IL-1β, and IL-6, promoting immune cell recruitment and antimicrobial responses.^53^, ^54^, ^55^, ^56^ These cells also contribute to antigen presentation and help initiate adaptive immunity.^57^ As infection resolves, macrophages shift toward a reparative phenotype that supports inflammation resolution and tissue repair.^10^ Neutrophils are rapidly recruited to infected tissues and eliminate pathogens through phagocytosis, release of antimicrobial enzymes, and production of reactive oxygen species (ROS).^58^, ^59^, ^60^, ^61^, ^62^ They can also generate neutrophil extracellular traps (NETs) that capture and restrict pathogens, thereby limiting their spread.^63^

In addition, pattern-recognition receptors (PRRs) on epithelial cells and APCs detect pathogen-associated molecular patterns (PAMPs) and activate IRF3/NF-κB pathways, leading to secretion of type I (IFN-α/β) and type III (IFN-λ) cytokines.^64^ These cytokines play a key role in the early confrontation of viral infections and modulate further adaptive immunity.^65^, ^66^, ^67^, ^68^

## Adaptive immunity in the respiratory mucosa

Antigen-mediated adaptive immunity is the final defense against pathogens, developing over days to weeks. The compact epithelial barrier limits passive macromolecule transport, while microfold cells (M cells) transport luminal antigens to underlying APCs via transepithelial pockets, providing a direct gateway for mucosal antigen sampling.^8^^,^^69^ In contrast, DCs rarely capture luminal antigens directly but extend transepithelial dendrites upon epithelial CCL20 chemotaxis and receptor interactions.^70^

**Figure 1:**
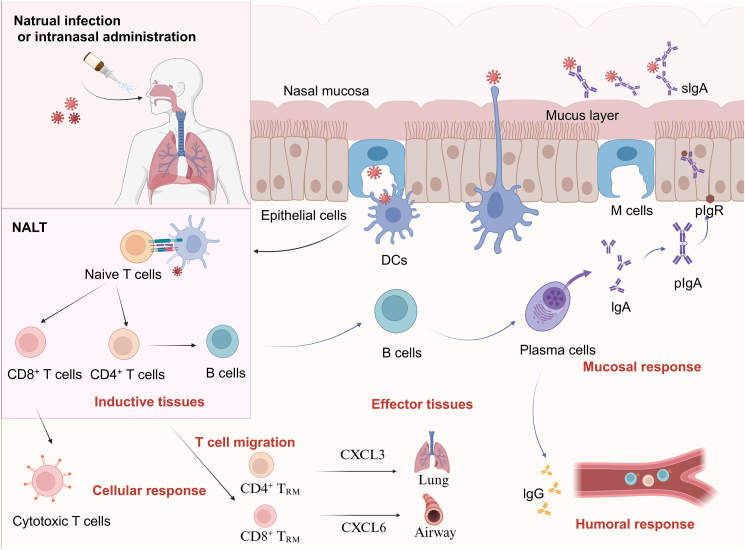
Adaptive immune responses in the RM immune system. Antigens penetrate the mucosal surface and are captured by M cells, specialized in antigen uptake in the NALT. These antigens are then transported to DCs for processing and presentation. Subsequently, DCs migrate to the interfollicular T-cell and follicular B-cell regions, where they present processed antigens to primed T cells, leading to their activation, differentiation, and expansion, ultimately triggering a range of adaptive immune responses, including CTL responses, systemic humoral immunity, mucosal SIgA production, and TRM cell formation. The scheme was generated using Biorender.

Activated DCs migrate to interfollicular regions to present peptide antigens to naïve T cells, driving differentiation into CD8^+^ cytotoxic and CD4^+^ helper subsets.^1^^,^^71^ Activated CD4^+^ helper T cells provide essential signals, including CD40–CD40L interaction and cytokines such as IL-21, IL-4, and TGF β, that drive B cell activation, affinity maturation, and immunoglobulin class switching within lymphoid follicles.^72^^,^^73^ Tissue-specific homing receptors—integrins and chemokine receptors (CCRs)—guide effector lymphocytes to airway sites where B cells mature into plasma cells secreting IgG for systemic or IgA for mucosal protection.^74^ T cells migrate into the airway lumen, along the walls of large bronchi, and within the pulmonary parenchyma surrounding bronchioles and alveoli, where they differentiate into TRM cells, enabling rapid recall responses to reinfection (Fig. 1).^75^^,^^76^

SIgA dominates respiratory surfaces (∼70% of total immunoglobulin) and is efficiently induced by intranasal, rather than intramuscular vaccination.^77^ Class-switched IgA^+^ B cells exit mucosa-associated lymphoid tissue (MALT) via lymphatics and, guided by CCR10–CCL28 signaling, home to distal mucosal sites.^33^^,^^78^ At effector sites, they differentiate into plasma cells secreting monomeric IgA, which polymerizes via the J-chain and binds to the poly-Ig receptor (pIgR) for transcytosis.^79^^,^^80^ Cleavage of pIgR generates SIgA on the epithelial surface, providing neutralization and immune exclusion.^81^

Collectively, the RM immune system achieves effective surveillance and pathogen clearance through the integrated actions of phagocytes, IFNs, CD8^+^ cytotoxic T cells, and pathogen-specific antibodies.

## Tissue repair and regeneration

Following pathogen clearance, the respiratory mucosa transitions from an inflammatory to a reparative phase aimed at restoring epithelial integrity and barrier function. M2 macrophages represent a key component of the regenerative niche after airway epithelial injury. These cells secrete high levels of anti-inflammatory cytokines such as IL-10 and TGF-β, which help suppress inflammatory responses and promote tissue repair. M2 macrophages further contribute to the synthesis and remodeling of extracellular matrix components, including collagen, fibronectin, and proteoglycans, thereby facilitating structural recovery.^10^^,^^82^ Simultaneously, IL-22, produced by both innate and adaptive immune cells, drives the proliferation of epithelial stem cells and the reorganization of tight junctions, thereby enhancing mucosal defense against secondary injury.^44^^,^^83^ ILC3 subsets also serve as key mediators in mucosal protection and repair during infection, primarily through the production of IL-17 and IL-22.^82^^,^^84^

## Immunological memory and long-term protection

After pathogen clearance, the RM immune system establishes durable immune memory through the coordinated activity of LLPCs, MBCs, and TRM cells. These components integrate humoral and cellular immunity to provide rapid protection against reinfection.

## Long-lived plasma cells and memory B cells

Durable humoral protection depends on two major B-cell populations. LLPCs continuously secrete antibodies independently of antigen stimulation, while MBCs rapidly re-enter the plasma-cell pathway upon re-exposure.^11^

The bone marrow serves as the primary survival niche for LLPCs, where plasma cells persist within specialized microenvironments that provide survival signals such as proliferation-inducing ligand (APRIL), IL-6, and BAFF.^85^^,^^86^ Notably, mucosal immunity in the respiratory mucosa can induce the generation of LLPCs not only in the bone marrow but also in mucosal sites such as NALT, where it promotes the formation of long-term IgA^+^ antibody-secreting cells (ASCs).^87^^,^^88^ The long-term colonization of IgA^+^ ASCs within NALT holds significant biological importance. Research indicates that these cells can directly secrete IgA antibodies into the acinar lumen, thereby establishing highly efficient local immune defenses.^33^ This mechanism confers key physiological advantages: firstly, serum antibodies struggle to permeate this region effectively.^89^ More critically, the directed recruitment and prolonged residency of effector plasma cells at infection sites foster high-concentration antibody microenvironments, thereby achieving potent local immune protection.^88^^,^^90^^,^^91^

Unlike MBCs in systemic circulation, RM MBCs can colonize local tissues, persisting long-term in the lungs and upper airways.^92^^,^^93^ They express unique mucosal homing receptors (such as CCR9, CCR10, and α4β7 integrin), enabling localization to subepithelial regions.^94^^,^^95^ These cells remain quiescent under homeostatic conditions but can be rapidly activated upon re-exposure to pathogens by local APCs or epithelial-derived cytokines (such as BAFF and APRIL).^96^ They then differentiate into plasma cells, secreting high-affinity IgA and IgG antibodies to mediate a rapid and robust secondary immune response.^92^^,^^97^ Following respiratory infections caused by influenza viruses, SARS-CoV-2, respiratory syncytial virus (RSV), and others, locally formed MBCs can persist for months to years.^98^ Research confirms that these tissue-resident MBCs exhibit superior persistence compared to circulating B cells, capable of generating high levels of secretory antibodies within extremely short timeframes upon re-exposure.^96^ This effectively blocks viral attachment and restricts its spread. Moreover, tissue-resident IgA^+^ MBCs may constitute the primary source of cross-protective antibodies. They can be rapidly activated during heterologous viral challenges and secrete cross-reactive IgA antibodies independently of T-cell help—a capability typically absent in plasma cells that have differentiated into high-yield, high-affinity ASCs.^92^^,^^93^^,^^99^

## Tissue-resident memory T cells

TRM cells are positioned within mucosal barriers to deliver immediate antigen-specific protection. In the respiratory tract, CD8^+^ TRM mediate cytolytic and IFN-γ–driven responses, whereas CD4^+^ TRM support CD8^+^ TRM persistence and augment B-cell immunity.^75^^,^^89^^,^^100^

Following a respiratory infection, antigens presented by DCs stimulate the maturation of naïve T cells within the draining lymph nodes. Then, effector T cells migrate from the lymph nodes to the URT or lungs, with concomitant high expression of transcription factors such as Blimp1, Hobit, Runx3, Bhlhe40, and Notch, along with downregulation of KLF2, T-bet, Eomes, and TCF1, leading to the TRM cells’ signature protein CD69 upregulation.^75^^,^^76^ CD69 acts as an antagonist of S1PR1, preventing TRM cells from leaving the tissue. In some tissues, TRM cells express the integrins CD103 (αEβ7) and CD49a, which facilitate their retention through interactions with epithelial E-cadherin and extracellular matrix collagen, respectively.^76^^,^^100^ In the respiratory mucosa, TRM cells also express the chemokine receptor CXCR3, enabling their migration into the lungs and confinement by local gradients of chemotactic ligands such as CXCL9, CXCL10, and CXCL11. In addition, TRM cells express CXCR6 to further facilitate their migration to the airways (Fig. 1).^76^^,^^101^

Functionally, TRM cells can mediate protection even without neutralizing antibodies.^15^ Beyond their canonical helper functions, CD4^+^ TRM cells can mediate potent, IFN-γ–dependent protection against influenza infection in the lung through intrinsic effector mechanisms independent of B cells and CD8^+^ T cells, highlighting their potential as valuable targets for the development of universal respiratory vaccines.^102^ Moreover, IL-17-producing nasal CD4^+^ TRM can recruit NET-active neutrophils for rapid clearance of *Bordetella pertussis*.^103^ These collective findings underscore the superior potential of TRM cells as a target for vaccines against respiratory pathogens.

Collectively, LLPCs, MBCs, and TRM cells form an integrated memory network that supports long-term mucosal and systemic protection against respiratory pathogens.^104^ In addition, RM immunity can influence immune responses at distal mucosal sites, contributing to broader systemic immune coordination.^34^^,^^105^^,^^106^

## Diseases related to respiratory mucosal immunity

The RM immune system not only maintains epithelial integrity and immune homeostasis under steady-state conditions but also plays a central role in protecting the host from invading pathogens while preventing excessive inflammation.^4^ Viral, bacterial, and chronic inflammatory disorders are intricately intertwined with mucosal immunity: inadequate barrier protection or immune responses render the host more susceptible to infection, whereas exaggerated or uncontrolled inflammation may result in epithelial injury, barrier disruption, and chronic pathological remodeling.^107^, ^108^, ^109^ Distinct from conventional reviews that focus on systemic inflammation or structural pathology, this section approaches respiratory diseases from the perspective of mucosal immunity, delineating its key mechanisms in antiviral and antibacterial defense and its pathological and therapeutic implications in chronic airway inflammation.

## Viral infections

Respiratory viruses, including influenza virus, RSV, and SARS-CoV-2, represent major challenges to mucosal integrity. The coordinated action of the ciliated epithelium, mucus layer, and SIgA forms a tripartite physical, chemical, and immunological barrier that intercepts viral particles and restricts invasion. Pattern recognition receptors such as RIG-I, MDA5, and TLRs in epithelial and innate immune cells sense viral RNA intermediates and trigger type I and type III IFN cascades, which in turn activate JAK-STAT signaling to induce ISGs that restrict viral replication.^4^

Beyond this classic antiviral role, IFNs also reshape the mucosal immune landscape. Type I IFNs enhance antigen presentation and cytotoxic T-cell activation, while type III IFNs act locally at epithelial surfaces to control viral spread without excessive inflammation.^107^^,^^108^ In parallel, the IL-17 axis supports antiviral defense by promoting neutrophil recruitment, inducing epithelial production of mucins and AMPs, and facilitating barrier repair. Moderate IL-17 responses complement IFN-mediated restriction of viral replication, whereas uncontrolled IL-17 activation amplifies immunopathology and tissue damage.^110^^,^^111^ In parallel, the IL-17 axis supports antiviral defense by promoting neutrophil recruitment, inducing epithelial production of mucins and AMPs, and facilitating barrier repair. Moderate IL-17 responses complement IFN-mediated restriction of viral replication, whereas uncontrolled IL-17 activation amplifies immunopathology and tissue damage.^14^

Vaccination remains the cornerstone of defense against respiratory viruses. Conventional systemic immunization primarily elicits circulating immune responses but often fails to establish effective protection at the RM surface. In contrast, mucosal vaccines delivered intranasally or by inhalation activate local immunity directly at the site of viral entry, inducing pivotal effector components such as SIgA and TRM cells. These responses build a robust frontline barrier that complements systemic immunity. This strategy has demonstrated great promise for the prevention of influenza and SARS-CoV-2, and represents an important future direction for combating emerging respiratory viral threats.^15^^,^^17^

## Bacterial and fungal infection

Bacterial pathogens, including *Mycobacterium tuberculosis*, *Streptococcus pneumoniae*, *Klebsiella pneumoniae*, and *Haemophilus influenzae*, exploit weaknesses in mucosal defense to colonize the airways. The epithelial-mucus-SIgA axis functions cooperatively to entrap and neutralize bacteria: ciliary clearance removes immobilized microbes, while SIgA prevents adhesion to epithelial receptors. Airway epithelial cells also secrete lysozyme, lactoferrin, and AMPs that provide immediate bactericidal activity. The IL-17/Th17 pathway plays a crucial role in clearing extracellular bacterial infections, which orchestrates neutrophil recruitment and activation, enhancing bacterial clearance via phagocytosis and the formation of NETs.^103^ However, sustained or excessive Th17 responses can damage epithelial integrity and exacerbate inflammation.^103^ Bacteria simultaneously evolve multiple immune evasion mechanisms. For example, *Streptococcus pneumoniae* secretes exoglycosidases and IgA1 protease, which degrade host mucin glycans and cleave IgA1 molecules, thereby weakening mucosal antibody-mediated protection and facilitating bacterial adhesion, biofilm formation, and immune evasion. In addition, the polysaccharide capsule—a major virulence factor—impairs phagocytosis and limits complement activation, representing a key target in current pneumococcal vaccine design.^112^ The respiratory microbiome also plays a pivotal role in mucosal defense. A balanced microbial community contributes to colonization resistance, whereas dysbiosis, marked by reduced α-diversity and enrichment of opportunistic species, disrupts this barrier and alters immune thresholds through microbial metabolites and molecular patterns.^113^ Previous studies indicate that asymptomatic colonization or mucosal immunization can induce cross-reactive humoral and cellular immunity, offering protection against invasive bacterial pneumonia and supporting the rationale for mucosa-targeted vaccination strategies.^114^

Notably, the functions of IFNs and IL-17 extend beyond antiviral defense. IL-17 is indispensable for antifungal immunity, particularly against Candida albicans, by promoting neutrophil recruitment and strengthening epithelial barriers.^115^^,^^116^ Likewise, type I IFNs can modulate antifungal responses and influence the outcome of co-infections.^117^ This is clinically relevant, as COVID-19 patients frequently suffer from secondary fungal infections, where the balance of these cytokine pathways may determine whether fungal co-infections are resolved or exacerbated.^118^ For instance, in COVID-associated pulmonary aspergillosis (CAPA), a robust type I IFN response is induced, which paradoxically exacerbates immunopathological damage in severe COVID-19 patients.^108^ Thus, a brief consideration of these interactions highlights the broader immunological significance of IFNs and IL-17, linking antiviral and antifungal defenses and emphasizing their role in viral–fungal co-morbidities.

## Chronic inflammatory diseases

Chronic inflammatory respiratory diseases, including asthma, COPD, and cystic fibrosis (CF), are characterized not only by structural barrier disruption but, more critically, by profound dysregulation of RM immunity. This section focuses on how aberrant interplay between mucosal immune cells (e.g., ILCs, Th17 cells), cytokines, and effector molecules (e.g., SIgA) drives disease pathology and highlights their potential as therapeutic targets.^119^

Asthma represents a paradigm of maladaptive mucosal immunity, predominantly driven by type 2 inflammation.^120^ While epithelial barrier defects allow allergen entry, it is the subsequent dysregulation of the epithelial–immune axis that perpetuates disease.^121^ Epithelial-derived alarmins (IL-25, TSLP, and IL-33) act as master regulators, activating ILC2s and Th2 cells to orchestrate a broad type 2 inflammatory response.^109^^,^^122^^,^^123^ Activated ILC2s and Th2 cells secrete high levels of IL-4, IL-5, and IL-13, which collectively initiate eosinophilic inflammation, IgE class switching, and mucus hypersecretion.^124^, ^125^, ^126^, ^127^ The therapeutic validation of this pathway is evidenced by the clinical success of biologics targeting TSLP (tezepelumab) and IL-4Rα (dupilumab), which effectively block this mucosal inflammatory cascade.^128^^,^^129^ Beyond cytokines, the SIgA system is compromised in asthma. Th2 cytokines, particularly IL-4 and IL-13, downregulate epithelial pIgR expression, leading to local SIgA deficiency.^130^ This impairment of “immune exclusion” facilitates allergen sensitization and viral exacerbations, identifying the restoration of the pIgR/SIgA axis as a potential strategy to reinforce the mucosal shield.^131^^,^^132^

COPD pathology is driven by a shift toward type 1 and type 3 mucosal immunity, distinct from the type 2 bias in asthma. Elevated ILC1 frequency correlates with disease severity, and cigarette smoke—a major risk factor for COPD—can drive ILC2 to ILC1 conversion via IL-1β and IL-12 signaling.^133^^,^^134^ Besides, IL-17-secreting ILC3 cells may play a pivotal role in COPD pathogenesis, with the central role of IL-17A in regulating inflammatory responses in chronic respiratory diseases.^135^, ^136^, ^137^ Airway epithelial expression of secretory components, derived from pIgR cleavage, is markedly reduced in both the large and small airways of COPD patients, correlating with airflow limitation and neutrophilic infiltration.^138^^,^^139^ Consistent with this, pIgR-deficient animal models develop spontaneous COPD-like inflammation through SIgA-microbiota dysregulation. Therapeutically, modulation of IL-17 signaling or restoration of the pIgR/SIgA barrier offers the potential to attenuate mucosal inflammation and slow disease progression.^140^^,^^141^

CF, in contrast, is an autosomal recessive genetic disorder caused by variants in the cystic fibrosis transmembrane conductance regulator (CFTR) gene. Although CF is not primarily an inflammatory disease, defective CFTR function leads to epithelial barrier dysfunction, mucus dehydration, and impaired mucociliary clearance, which secondarily promote chronic infection and persistent airway inflammation.^142^ Recent studies implicate ILC2s and Th17-associated cytokines in CF mucosal immune dysregulation: IL-17A–producing ILC2s from CF nasal polyps induce epithelial IL-8 secretion and neutrophil recruitment, while reduced circulating CCR6^+^ ILC2s are associated with worse lung function.^143^^,^^144^ Notably, CF mucosa exhibits altered pIgR/SIgA regulation compared with asthma and COPD. Enhanced epithelial pIgR expression and elevated IgA levels have been observed in lung transplant specimens from CF patients, and the upregulation of pIgR via IL-17 signaling is replicated in F508del mouse models.^145^ Increased serum and bronchoalveolar IgA may reflect compensatory mucosal immune activation, yet reduced IgA secretion at other mucosal sites (saliva, gastric fluid) suggests compartment-specific regulation.^146^^,^^147^ Targeting excessive IL-17-mediated responses or normalizing site-specific IgA homeostasis may offer innovative immunomodulatory strategies for CF.

## Prevention and therapy based on respiratory mucosal immunity

The growing burden of respiratory infections and inflammatory airway diseases has driven the development of preventive and therapeutic strategies targeting RM immunity. Among these, inhalable antibody formulations and RM vaccines have emerged as two of the most promising translational approaches.^19^^,^^148^^,^^149^ Below, we summarize recent progress and key challenges in their clinical development.

## Advances in inhalable antibodies

Inhalable antibodies represent an emerging strategy in RM immunotherapy, designed to deliver potent neutralizing antibodies or their functional fragments (such as single-chain variable fragments or nanobodies) directly to the nasal–bronchial–alveolar interface via aerosolization or intranasal administration. This localized delivery establishes a high-concentration immune barrier at the earliest site of infection, enabling rapid viral neutralization, reduction of pathogen burden, and minimization of systemic exposure-related side effects and manufacturing costs.^19^^,^^150^

Preclinical and early clinical studies have demonstrated that inhaled antibodies achieve substantially higher local concentrations in the lung and nasal mucosa than intravenously administered counterparts, leading to early suppression of viral replication and attenuation of tissue pathology.^151^, ^152^, ^153^, ^154^ Several inhalable antibody candidates against SARS-CoV-2 have shown robust efficacy in animal models, including the IgM-derived monoclonal antibody 1212C2, the human single-chain variable fragment 76clAb, and the bispecific single-domain antibody bn03.^151^, ^152^, ^153^ Moreover, the trimeric anti-RSV nanobody ALX-0171, evaluated in a randomized controlled clinical trial, demonstrated delivery feasibility and antiviral activity despite limited clinical improvement.^155^ In addition, a single intranasal dose of the neutralizing antibody 35B5 provided approximately 24-h protection in healthy volunteers against major SARS-CoV-2 variants of concern (Alpha, Beta, Delta, and Omicron).^154^

Despite these encouraging advances, no antibody therapy has yet been approved by any regulatory authority for administration via the inhalation route.^19^ The path toward clinical translation remains challenged by variant-dependent affinity differences, formulation instability under nebulization, device–formulation compatibility, and the potential risk of antibody-dependent enhancement (ADE).^156^ Antibody cocktail therapies may mitigate variant escape to some extent.^157^ In addition, maintaining the durable activity of inhalable antibodies and improving delivery systems should be considered.^156^ Notably, the Fc domain of conventional antibodies exerts a dual role—enhancing antiviral responses through immune engagement but also posing a risk of ADE.^150^^,^^158^ In contrast, nanobodies, as naturally occurring heavy-chain-only antibodies devoid of Fc domains, offer a promising and safer alternative that combines high aerosol stability with efficient mucosal delivery while circumventing ADE-associated risks.^159^^,^^160^

## Advances in respiratory mucosal vaccines

Vaccine development remains central to respiratory disease prevention. Although intramuscular vaccines effectively reduce severe LRT disease by inducing systemic immunity, they often provide limited protection at the mucosal portal of pathogen entry and therefore may not fully prevent infection or transmission.^78^^,^^161^, ^162^, ^163^ In contrast, RM vaccination can induce both local and systemic immune responses and is therefore considered a promising strategy against pathogens such as SARS-CoV-2, influenza virus, and RSV that initiate infection at the airway surface.^164^, ^165^, ^166^

**Figure 2:**
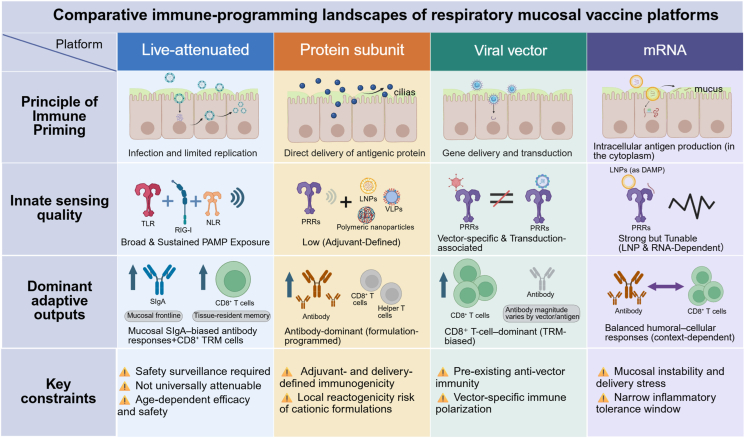
Immune-programming features of respiratory mucosal vaccine platforms. This schematic compares four major RM vaccine platforms—live-attenuated, protein subunit, viral vector and mRNA–LNP vaccines—highlighting differences in epithelial engagement, innate sensing, dominant adaptive outputs and key translational constraints. Live-attenuated vaccines partially mimic natural infection through limited mucosal replication, promoting broad innate activation and favoring SIgA and CD8^+^ TRM cells induction, but require stringent safety control. Protein subunit vaccines rely on purified antigen delivery, often with nanoparticle or VLP formulations; innate activation is typically adjuvant dependent and responses are commonly antibody dominant, with immunogenicity and reactogenicity shaped by formulation design. Viral vector vaccines drive antigen expression via cellular transduction, with vector-specific innate sensing and a frequent bias toward CD8^+^ T cell responses; pre-existing anti-vector immunity represents a key limitation. mRNA-LNP vaccines enable endogenous antigen production and tunable innate activation, but their effectiveness in mucosal settings depends on delivery efficiency, inflammatory tolerance and formulation stability. The scheme was generated using Biorender.

RM vaccines can be administered intranasally to target the URT or by aerosol inhalation to extend delivery to the lower airways.^167^^,^^168^ A variety of platforms, including live-attenuated, inactivated, viral vector, protein subunit, and mRNA vaccines, are currently being explored, and several candidates targeting RSV, Mycobacterium tuberculosis, influenza virus, Bordetella pertussis, and SARS-CoV-2 have advanced to clinical evaluation.^78^^,^^169^ Table 1 summarizes representative clinical RM vaccine candidates, and Figure 2 outlines the major immune-programming features of different platforms.

**Table 1:** Clinical progress in the development of intranasal vaccines for the common respiratory infectious diseases.

Pathogen	Vaccine candidates	Platform	Developers	Sponsor	Dose	Schedule	Phase	Trial registries
RSV	RSVt vaccine	LAV		Sanofi Pasteur, a Sanofi Company	2	Day 0 + 56	III	NCT06252285 NCT06705140
	MV-012-968	LAV		Meissa vaccines, Inc.	1	Day 0	II	NCT04690335
	MEDI-534	LAV		MedImmune LLC	3	Day 0 + 56 + 112	I/II	NCT00686075
	MEDI-559	LAV		MedImmune LLC	3	Day 0 + 56 + 112	I/II	NCT00767416
	BLB-201	LAV	Blue Lake Biotechnology Inc.	Blue Lake Biotechnology Inc.	1	Day 0	I/II	NCT05655182
							I	NCT05281263
	RSV vaccine formulation 1	LAV		Sanofi Pasteur, a Sanofi Company	1–2	Day 0 or day 0 + 56	I/II	NCT04491877
	SeVRSV	VVr	Cincinnati Children’s Hospital Medical Center, Cincinnati, OH (CCHMC)	National Institute of Allergy and infectious diseases (NIAID)	1	Day 0	I	NCT03473002
	PanAd3-RSV	VVnr	Advent, Italy		1	Day 0	I	NCT01805921
	MVA-BN-RSV	VVnr		Bavarian Nordic	1	Day 0	I	NCT02864628
	SynGEM	VVnr		Mucosis BV	2	Day 0 + 28	I	NCT02958540
	RSV ΔNS2/Δ1313/I1314L/RSV 276	LAV	Charles River Laboratories, Malvern, PA	National Institute of Allergy and infectious diseases (NIAID)	1	Day 0	I	NCT03227029 NCT03422237 NCT01893554 NCT03916185
	D46/NS2/N/ΔM2-2-HindIII	LAV	Charles River Laboratories, Malvern, PA	National Institute of Allergy and infectious diseases (NIAID)	1	Day 0	I	NCT03102034 NCT03099291
	RSV LID ΔM2-2 1030s vaccine	LAV	Charles River Laboratories, Malvern, PA	National Institute of Allergy and infectious diseases (NIAID)	1	Day 0	I	NCT02794870 NCT02952339 NCT04520659
	RSV 6120/ΔNS2/1030s vaccine	LAV	Charles River Laboratories, Malvern, PA	National Institute of Allergy and infectious diseases (NIAID)	1	Day 0	I	NCT03387137 NCT03916185
	RSV 6120/ΔNS1	LAV		National Institute of Allergy and infectious diseases (NIAID)	1	Day 0	I	NCT03596801
	RSV 6120/F1/G2/ΔNS1	LAV		National Institute of Allergy and infectious diseases (NIAID)	1	Day 0	I	NCT03596801
	RSVcps2	LAV		National Institute of Allergy and infectious diseases (NIAID)	1	Day 0	I	NCT01968083 NCT01852266
	RSV MEDI ΔM2-2 vaccine	LAV	Charles River Laboratories, Malvern, PA	National Institute of Allergy and infectious diseases (NIAID)	1	Day 0	I	NCT02237209 NCT02040831
	RSV LID ΔM2-2 vaccine	LAV		National Institute of Allergy and infectious diseases (NIAID)	1	Day 0	I	NCT01459198
	RSV LID cp ΔM2-2 vaccine	LAV	Charles River Laboratories, Malvern, PA	National Institute of Allergy and infectious diseases (NIAID)	1	Day 0	I	NCT02890381 NCT02948127
	CodaVax-RSV	LAV		Codagenix, Inc.	1	Day 0	I	NCT04919109
*M. tuberculosis*	ChAdOx1 85A	VVnr	Clinical Biomanufacturing Facility, University of Oxford, Oxford, UK	François Spertini	1	Day 0	I	NCT04121494
	AdHu5Ag85A	VVnr	Robert E. Fitzhenry Vector Laboratory, McMaster Immunology Research Center, McMaster University	McMaster University	1	Day 0	I	NCT02337270
	MVA85A	VVnr	IDT Biologika, Dessau-Rosslau, Germany	University of Oxford	1	Day 0	I	NCT01497769 NCT02532036
	Ag85B-ESAT6 fusion protein H1	PS		St George’s, University of London	2	Day 0 + 56	I	NCT00440544
	BCG Danish	LAV		University of Oxford	1	Day 0	I	NCT04777721
Influenza	SIIL LAIV	LAV		PATH	1	Day 0	III	NCT01854632
	CAIV-T	LAV	Wyeth, Marietta, PA, USA	MedImmune LLC	1	Day 0	III	NCT00192413
	NasoVAX	VVnr	Emergent Manufacturing Operations in Baltimore	Altimmune, Inc.	1	Day 0	II	NCT04442230
	Flucelvax(R)	IV		National Institute of Allergy and infectious diseases (NIAID)	1	Day 0	II	NCT03845231
	GHB16L2	LAV		AVIR Green Hills Biotechnology AG	1	Day 0	I/II	NCT01369862
	Vaccine A/17/CA/2009/38 (H1N1)	LAV		Mahidol University	1	Day 0	I/II	NCT01666262
	Vaccination	PS		Hadassah Medical organization	1	Day 0	I/II	NCT00197301
	AdhVN1203/04.H5	VVnr	University of Alabama at Birmingham	Altimmune, Inc.	2	Day 0 + 28	I	NCT00755703
	Ad4-H5-Vtn	VVr	Emergent Biosolutions Inc.	National Institute of Allergy and infectious diseases (NIAID)	1	Day 0	I	NCT01806909
	Trivalent inactivated influenza virus vaccine	IV		National Institute of Allergy and infectious diseases (NIAID)	1	Day 0	I	NCT00436046
	BPL-1357	IV		National Institute of Allergy and infectious diseases (NIAID)	1	Day 0	I	NCT05027932
	cH8/1N1 LAIV	LAV	PATH	PATH	1	Day 0	I	NCT03300050
	LAIV H7N3	LAV	PATH	PATH	2	Day 0 + 28	I	NCT01511419
	H2N3 MO 2003/AA ca vaccine	LAV		National Institute of Allergy and infectious diseases (NIAID)	2	Day 0 + 28	I	NCT01175122
	Live-attenuated H7N9 A/Anhui/13 ca	LAV		National Institute of Allergy and infectious diseases (NIAID)	1	Day 0	I	NCT02251288
					1–2	Day 0 or 0 + 28	I	NCT01995695 NCT02151344
	Live influenza A vaccine H7N3 (6-2) AA ca	LAV		National Institute of Allergy and infectious diseases (NIAID)	2	Day 0 + 28-56	I	NCT00516035 NCT00853255
	H9N2 (6-2) AA ca recombinant vaccine	LAV	Novavax	National Institute of Allergy and infectious diseases (NIAID)	1	Day 0	I	NCT00110279
					2	Day 0 + 28-84	I	NCT00380237
	H2N2 1960 AA ca recombinant vaccine	LAV		National Institute of Allergy and infectious diseases (NIAID)	2	Day 0 + 28-62	I	NCT00722774
	H6N1 Teal HK 97/AA ca recombinant vaccine	LAV		National Institute of Allergy and infectious diseases (NIAID)	2	Day 0 + 28-62	I	NCT00734175
	Influenza A H7N7 vaccine	LAV		National Institute of Allergy and infectious diseases (NIAID)	2	Day 0 + 28-62	I	NCT00922259
	H5N1 (6-2) AA ca recombinant vaccine	LAV		National Institute of Allergy and infectious diseases (NIAID)	2	Day 0 + 28-56	I	NCT00488046
	OVX836	PS		Osivax	2	Day 0 + 28	I	NCT03594890
	BW-1014	PS		BlueWillow biologics	2	Day 0 + 28	I	NCT05397119
*B. pertussis*	BPZE1	LAV	Boostrix, GlaxoSmithKline, London, UK	ILiAD Biotechnologies	1	Day 0	II	NCT03942406 NCT03541499 NCT05116241 NCT05461131
	Vaccine GamLPV	LAV		Gamaleya research Institute of Epidemiology and Microbiology, health Ministry of the Russian Federation	1	Day 0	I/II	NCT04036526
							I	NCT03137927
SARS-CoV-2	Ad5-nCoV	VVnr	CanSino Biologics, Tianjin, China	Jiangsu Province Centers for disease control and prevention	1	Day 0	IV	NCT05303584
			Convidecia, CanSino	CanSino biologics Inc.			III	NCT05517642
	BBV154	VVnr	Bharat Biotech, Hyderabad, India	Bharat Biotech International limited	2	Day 0 + 28	III	NCT05522335
								CTRI/2022/02/40065
	DelNS1-2019-nCoV-RBD-OPT1	VVr	University of Hong Kong, Xiamen University and Beijing Wantai Biological Pharmacy	Beijing Wantai biological Pharmacy Enterprise	2	Day 0 + 14	III	ChiCTR2100051391
				The University of Hong Kong	2	Day 0 + 21	II	NCT05200741
	COVI-VAC	LAV	Codagenix, NewYork, USA and SerumInstitute, Pune, India	Codagenix; serum Institute of India	1–2	Day 0 or 0 + 28	III	ISRCTN15779782
	HH-120	PS		Huahui health	8	Day 0 + 1 + 2 + 3 + 4 + 5 + 6 + 7	III	NCT05787418
	AVX/COVID-12	IV	Laboratorio Avi-Mex, S.A. de C.V.	Laboratorio Avi-Mex, S.A. de C.V.	1	Day 0	II	NCT05205746
	ARCoV	RNA vaccine	Academy of Military Science (AMS), Walvax Biotechnology and Suzhou Abogen Biosciences	Yunnan Walvax Biotechnology	1	Day 0	II	ChiCTR2100041855
	Razi Cov pars	PS	Razi Vaccine and Serum Research Institute	Razi vaccine and serum research Institute	3	Day 0 + 21 + 51	II	IRCT20201214049709N2
	Gam-COVID-Vac	VVnr	Gamaleya NRCEM, Moscow, Russia	Gamaleya research Institute of Epidemiology and Microbiology, health Ministry of the Russian Federation	1	Day 0	I/II	NCT05248373
	CIGB-669	PS	Center for Genetic Engineering and Biotechnology (CIGB)	Center for genetic engineering and Biotechnology (CIGB)	3	0 + 14 + 28 or 0 + 28 + 56	I/II	RPCEC00000345
	AdCOVID	VVnr		Altimmune, Inc.	1	Day 0	I	NCT04679909
	CVXGA1	VVnr	CyanVac and Blue Lake Biotechnology	CyanVac LLC	1	Day 0	I	NCT04954287
	SC-Ad6–1	VVnr	Tetherex Phar	Moat Biotechnology Corporation	1–2	Day 0 or 0 + 28	I	NCT04839042
	AZD1222/ChAdOx1 nCov-19	VVnr	University of Oxford and AstraZeneca Biopharmaceuticals	University of Oxford	1–2	Day 0 or 0 + 28	I	NCT04816019
	Ad5-triCoV/Mac, Chad-triCoV/Mac	VVnr	McMaster University	McMaster University	1	Day 0	I	NCT05094609
	MVA-SARS-2-ST vaccine	VVnr	Hannover Medical School	Hannover Medical School	1	Day 0	I	NCT05226390
	NDV-HXP-S	VVr	Laboratorio Avi-Mex, S.A. de C.V.	Laboratorio Avi-Mex, S.A. de C.V.	2	Day 0 + 21	I	NCT04871737
				Sean Liu	1	Day 0	I	NCT05181709
	MV-014-212	LAV	Meissa Vaccines, Inc.		1–2	Day 0 + 36	I	NCT04798001
	B/HPIV3/S–6P	LAV		National Institute of Allergy and infectious diseases (NIAID)	2	Day 0 + 56	I	NCT06026514
	LNP-nCoVsaRNA	RNA vaccine	Imperial College London	Imperial College London	2	Day 0 + 28	I	ISRCTN17072692
	ACM Biolabs ACM-SARS-CoV-2-beta ACM CpG vaccine candidate (ACM-001)	PS	ACM Biolabs	ACM Biolabs	1	Day 0	I	NCT05385991

VVnr: Viral Vector (non-replicating), VVr: Viral Vector (replicating), IV: Inactivated Virus, LAV: Live Attenuated Virus, PS: protein subunit.

Data from https://clinicaltrials.gov/, https://www.fda.gov/, and https://www.who.int.

## Live-attenuated vaccines and inactivated vaccines

Live-attenuated vaccines are based on pathogens that have reverse genetics or adaptation to reduce virulence, while inactivated vaccines are produced by chemically or thermally inactivating viruses *in vitro*. Inactivated vaccines use the whole viral particles as immunogens, preserving the structural integrity of the virus and thereby inducing a broader spectrum of antibody responses than subunit vaccines. However, inactivation processes may result in the loss of antigens or PAMPs, potentially diminishing the magnitude and durability of immune responses. In comparison, live-attenuated vaccines have the advantages of stimulating pathogen-associated PRRs, high immunogenicity without adjuvants, and better efficacy in overcoming epithelial barriers compared to antigen-only formulations.^170^ Furthermore, live-attenuated vaccines typically exhibit higher production yields during manufacturing processes than inactivated vaccines, making them particularly advantageous in developing countries due to their robust immune response and ease of administration.^171^

Intranasal vaccines currently approved for human use (excluding Emergency Use Authorization) are all based on live-attenuated influenza vaccines (Table 2). Although live-attenuated virus vaccines have irreplaceable advantages in RM immunity, several challenges limit their widespread application. Firstly, there is a risk associated with the use of live viruses, necessitating long-term safety monitoring. Secondly, not all viruses can be attenuated. Finally, live-attenuated vaccines may exhibit variable efficacy and safety profiles among different age groups, such as children, adults, and the elderly, requiring comprehensive evaluation across all target age groups.^170^ To address these limitations, innovative attenuation strategies, such as genetic codon deoptimization, have demonstrated promising potential in enhancing live-attenuated vaccine safety and efficacy.^172^

**Table 2:** Respiratory mucosal vaccines approved (including EUA) for human use against the respiratory virus.

Disease	Vaccine	Platform	Developers	Dosage form	Route of administration	Phase	Approval date
Influenza	FluMist	LAV	MedImmune, LLC	Spray	IN	FDA-approved	2003
	FluMist Quadrivalent	LAV	MedImmune, LLC	Spray	IN	FDA-approved	2012
	Nasovac-S	LAV	Serum Institute of India Pvt. Ltd.	Freeze-dried	IN	CDSCO-approved	2010
	Fleunz Tetra	LAV	AstraZeneca	Spray	IN	EMA-approved	2013 (Withdrawn in 2025)
	Ganwu	LAV	Changchun BCHT Biotechnology Co.	Freeze-Dried	IN	NMPA-approved	2020
COVID19	Convidecia air	VVnr	CanSino Biologics Inc.	Liquid suspension	Aerosol delivery	NMPA-approved (EUA)	2022
	BBV154/iNCOVACC	VVnr	Bharat Biotech International limited	Spray	IN	CDSCO-approved (EUA)	2022
	DelNS1-2019-nCoVRBD-OPT1	VVnr	The University of Hong Kong, Xiamen University and Beijing Wantai Biological Pharmacy	Spray	IN	NMPA-approved (EUA)	2022
	Sputnik nasal	VVnr	The Gamaleya Research Institute of Epidemiology and Microbiology	Spray	IN	Minzdrav-approved (EUA)	2022
	Razi Cov Pars	PS	Razi Vaccine and Serum Research Institute	Spray	IN	IFDA-approved (EUA)	2021

EUA: Emergency Use Authorization; LAV: Live attenuated virus; VVnr: Viral vector (non-replicating); PS: Protein subunit; IN: Intranasal administration; FDA: Food and Drug Administration; CDSCO: Central Drugs Standard Control Organization; EMA: European Medicines Agency; NMPA: National Medical Products Administration; Minzdrav: Ministry of Health of the Russian Federation; IFDA: Iran Food and Drug Administration.

Data from https://clinicaltrials.gov/, https://www.fda.gov/, and https://www.who.int.

## Protein subunit vaccines

Protein subunit vaccines are based on protein or peptide fragments of target pathogens, which are produced using recombinant expression systems in insects, yeasts, bacteria, or mammalian cells.^173^, ^174^, ^175^, ^176^ Owing to their favorable safety profile, tolerability, and manufacturing controllability, they are widely viewed as safer and more predictable than whole-organism vaccines and have been explored extensively for respiratory pathogens.^177^ The emergency authorization of the protein-based COVID-19 vaccine Razi Cov Pars illustrates the translational feasibility of this platform (Table 2).

Despite these advantages, subunit vaccines face intrinsic challenges in RM vaccination. Soluble proteins are vulnerable to enzymatic degradation and mucociliary clearance and often provide insufficient innate activation for efficient APC engagement, limiting the induction of secretory IgA and durable tissue-localized memory.^177^^,^^178^ Consequently, effective RM subunit vaccines generally require adjuvants and delivery systems that enhance antigen stability, mucosal penetration/retention, and immune programming.

Nanotechnology-based formulations—lipid nanoparticles (LNPs), polymeric nanoparticles, and virus-like particles (VLPs)—are therefore central to RM subunit vaccine design. Liposome-based NPs are attractive for intranasal use because they are biocompatible and can co-deliver antigens with immunostimulatory molecules. Liposomes can be tuned (lipid composition, structure, and size) to match antigen properties, and cationic or mucoadhesive surface modifications can increase nasal residence.^179^ For example, in preclinical studies, an intranasal SARS-CoV-2 subunit formulation incorporating an LNP–monosodium urate adjuvant reduced airway viral loads and supported respiratory innate and adaptive immunity.^180^ Polymeric nanoparticles composed of natural or synthetic polymer offer additional versatility in tuning antigen release kinetics, surface charge, and mucosal adhesion. These parameters critically influence APC uptake and downstream immune polarization.^181^, ^182^, ^183^ Among these, cationic cholesteryl group pullulan (cCHP)-bearing nanogels are a promising nasal carrier that can improve mucosal retention and antigen delivery. cCHP-based formulations have supported mucosal IgA and systemic IgG responses and demonstrated protection in preclinical models against pneumococci and RSV challenges, highlighting their potential as modular RM subunit platforms.^184^^,^^185^ Besides, cationic carriers such as 1,2-Dioleoyl-3-trimethylammonium-propane (DOTAP), chitosan, and PEI can markedly enhance the immunogenicity of intranasally delivered SARS-CoV-2 receptor-binding domain (RBD) subunit vaccines, although highly cationic materials warrant careful evaluation of local reactogenicity and inflammatory risk to define an acceptable safety–efficacy window.^186^ Finally, particulate antigen-display approaches such as VLPs provide virus-mimetic, repetitive geometries that enhance the antiviral immune response without genetic material. VLP–spike strategies have improved protein-based SARS-CoV-2 and RSV vaccination.^187^^,^^188^

Collectively, these findings highlight that while protein subunit vaccines are inherently safe and flexible, their success as RM vaccines critically depends on the rational integration of adjuvants and delivery systems that can compensate for weak innate immune activation and mucosal instability. A major future challenge lies in identifying formulations that simultaneously enhance mucosal immunogenicity, maintain safety, and enable scalable manufacturing for clinical translation.

## Viral vector vaccines

Viral vector vaccines have long been regarded as promising platforms for RM immunization owing to their ability to express heterologous antigens in host cells and to induce potent humoral and cellular immune responses without the need for exogenous adjuvants. In contrast to conventional vaccine strategies that primarily focus on neutralizing antibody induction, viral vectors broaden the spectrum of immunogenicity by robustly activating cytotoxic T cells, helper T cells, and innate immune pathways.^189^

A wide range of viral vectors—including vesicular stomatitis virus, parainfluenza virus, measles virus, influenza virus, adenovirus (AdV), and poxvirus vectors—have been investigated for RM vaccination.^169^^,^^190^ Among these, AdV has emerged as one of the most extensively studied candidates because of its strong immunogenicity, genetic flexibility, and natural tropism for respiratory tissues.^191^^,^^192^ Several intranasal adenovirus-vectored COVID-19 vaccines have already entered clinical evaluation, demonstrating the feasibility of this approach (Table 2). However, a major limitation of AdV-based vaccines is the widespread pre-existing immunity to common human serotypes, such as AdHu5, which can compromise vaccine efficacy.^193^ Strategies to overcome this issue include the use of rare human serotypes, engineered vectors, or non-human adenoviruses such as chimpanzee adenoviruses, which exhibit lower seroprevalence in human populations.^169^^,^^194^, ^195^, ^196^

Beyond seroprevalence, the immunological characteristics of different vectors can strongly influence vaccine performance. Distinct vectors activate different innate sensing pathways and therefore drive divergent immune polarization profiles, highlighting the importance of vector selection according to the desired immune outcome.^197^^,^^198^ For instance, comparative immunization studies in mice using adenoviral and poxviral vectors encoding identical antigens have revealed vector-specific immunogenicity. Adenoviral vectors preferentially activate TLR2-mediated signaling and promote γδ T cell and natural killer (NK) cell activation. In contrast, modified vaccinia Ankara (MVA) vector vaccination primarily stimulates the type I IFN signaling pathway.^197^ These insights have stimulated interest in heterologous prime–boost strategies combining complementary vectors or integrating different delivery routes to optimize mucosal and systemic immunity.^191^^,^^199^, ^200^, ^201^, ^202^ In parallel, advances in multivalent antigen design, particularly within AdV-based platforms, further extend this strategy by broadening antigenic coverage. Multivalent AdV vaccines have demonstrated broader protection against diverse SARS-CoV-2 variants in animal models than monovalent formulations. This directly showcases the potential of integrating antigenic breadth with rational vector selection.^18^ This directly showcases the potential of integrating antigenic breadth with rational vector selection.

Despite decades of development, relatively few RM viral vector vaccines have advanced to late-stage clinical evaluation.^169^ Future research should focus on systematically comparing vector-specific immune responses, tissue tropism, durability of mucosal protection, and safety profiles in humans. Such comparative studies will be essential for identifying optimal vector platforms and guiding the rational design of next-generation RM vaccines. In addition to viral vectors, bacterial vectors such as Lactobacillus, Salmonella, and Listeria have also been extensively explored as vaccine delivery platforms.^203^^,^^204^ However, their long-term efficacy and scalability remain important questions for future investigation.

## mRNA vaccines

mRNA vaccines have revolutionized vaccinology, offering rapid manufacturability, high potency, and non-integrating expression.^205^ During the COVID-19 pandemic, systemic (intramuscular) mRNA vaccines such as BNT162b2 and mRNA-1273 provided robust systemic protection but only weak neutralizing activity in the respiratory mucosa.^15^^,^^162^^,^^206^ This shortcoming has fueled growing interest in developing mucosal mRNA vaccines administered by inhalation or intranasal routes.^207^^,^^208^

However, successful mucosal mRNA vaccination must overcome several barriers unique to the respiratory tract. Nuclease-rich secretions, rapid mucociliary clearance, and physical stresses introduced during aerosolization or spray-drying can substantially compromise mRNA stability and bioavailability.^209^ As a result, the development of effective delivery systems has become a central focus of mucosal mRNA vaccine research. Advances in biomaterials engineering have led to the emergence of LNPs and cationic polymer–based carriers as promising platforms capable of protecting mRNA, facilitating mucus penetration, and promoting cellular uptake in airway tissue studies.^210^^,^^211^ This strategy has demonstrated promising efficacy in preclinical studies.^212^^,^^213^ As the most clinically advanced mRNA delivery vehicles, LNP formulations critically determine mucus penetration and immune cell targeting. Particle size, surface charge, PEG-lipid density, and lipid architecture collectively regulate nanoparticle diffusion through the mucus layer and preferential transfection of epithelial cells versus APCs.^209^^,^^214^^,^^215^ Notably, a recent study systematically compared clinically approved ionizable lipids, including ALC-0315, SM-102, and MC3, with or without incorporation of the permanently cationic lipid DOTAP, for intranasal mRNA-LNP delivery. The authors found that although intranasal administration enabled efficient transfection of pulmonary epithelial and immune cells, it remained poorly immunogenic as a primary vaccination route, eliciting weaker immune responses than intramuscular injection and highlighting a decoupling between delivery efficiency and mucosal immunogenicity. In contrast, intranasal mRNA-LNPs showed greater potential as booster vaccines by efficiently recalling systemic and mucosal immune memory, an effect further enhanced by DOTAP incorporation. Elucidating how different LNP compositions behave and interact with the immune system across distinct tissue microenvironments *in vivo* is essential for establishing design rules that enable the rational optimization of mRNA-LNP platforms to achieve optimal protection against respiratory pathogens.^216^

Finally, successful respiratory mRNA vaccines must achieve a finely tuned balance between immune activation and local inflammatory tolerance. While insufficient innate immune sensing at mucosal surfaces may limit adaptive immune priming, excessive activation—particularly mediated by strongly cationic lipids or potent innate immune agonists—can provoke airway inflammation and compromise tissue integrity.^205^^,^^217^, ^218^, ^219^ Together, these findings provide a strong rationale for the continued development of nasal mRNA vaccines capable of inducing protective immunity in humans.

## Strategic principles for next-generation respiratory mucosal vaccines

Despite substantial progress in RM vaccine platforms, achieving durable protection at the respiratory portal of entry remains challenging.^220^ Therefore, next-generation RM vaccines should be guided not only by platform selection but also by clearly defined mucosal immune goals, broader antigen design, and translational safety considerations.^220^^,^^221^

## Prioritizing upper respiratory tract-localized immunity and appropriate mucosal immune programming

Next-generation RM vaccines should be designed to establish robust immunity in the URT, the primary site of pathogen entry and transmission.^15^ Accordingly, vaccine efficacy should be evaluated not only by systemic antibody responses, but also by URT-relevant endpoints such as nasal SIgA, mucosal neutralization, reduced viral shedding, and the establishment of tissue-resident immune memory.^3^^,^^15^ This conceptual shift reframes protection from systemic immunogenicity toward local sterilizing or transmission-blocking immunity, which is increasingly recognized as critical for controlling rapidly spreading respiratory pathogens.

Effective URT protection depends on coordinated mucosal immune programming rather than antibody magnitude alone.^1^^,^^222^, ^223^, ^224^ SIgA provides immediate frontline neutralization at the mucosal surface, whereas tissue-resident memory T and B cells enable rapid local recall responses.^81^^,^^104^^,^^225^ In addition, appropriately polarized Th1- and Th17-associated responses may further support antiviral or antibacterial clearance and reinforce epithelial barrier function.^222^ Achieving such immunity requires efficient antigen delivery to mucosal inductive sites despite epithelial tight junctions, mucociliary clearance, and enzymatic degradation. Strategies including mucoadhesive or permeation-enhancing formulations, as well as M-cell- or dendritic cell-targeting approaches, may improve antigen access and shape the quality of mucosal priming.^73^^,^^226^, ^227^, ^228^, ^229^, ^230^, ^231^, ^232^, ^233^

Importantly, the optimal immune program for URT protection is inherently pathogen specific. Integrating immune profiling data obtained before and after natural infection or vaccination can help identify the immune modalities most relevant for protection against a given respiratory pathogen—whether humoral immunity, cellular responses, tissue-resident memory, or circulating memory, alone or in combination—and thereby guide rational RM vaccine design.^78^^,^^234^ Collectively, these considerations underscore a central principle for next-generation RM vaccines: durable protection at the respiratory portal of entry is best achieved through integrated immune engineering, in which barrier navigation, targeted antigen delivery, and deliberate immune programming are coordinated to induce transmission-limiting mucosal immunity.

## Upgrading antigen strategies toward breadth, preparedness, and multivalence

The development of pan-family or universal vaccines capable of conferring broad protection against all members of a viral family represents a central strategy in next-generation vaccine design.^235^ Previous efforts—particularly in influenza and Henipavirus vaccines—have established key conceptual and technical foundations for this approach.^236^^,^^237^ For coronaviruses, the urgency is particularly evident: three major global outbreaks caused by distinct coronaviruses have occurred within the past two decades, underscoring the high likelihood of future emergence events and the inherent limitations of reactive, pathogen-specific vaccine development.^238^ In contrast to this long-term preparedness goal, currently licensed SARS-CoV-2 vaccines largely follow a narrow-spectrum strategy. These vaccines encode strain-specific spike glycoproteins and predominantly elicit species- or even variant-restricted immune protection.^239^ While highly effective at preventing severe disease, such approaches require continual antigen updating and are inherently vulnerable to antigenic drift and immune escape. Broadly protective vaccines aim to overcome these limitations by shifting the design paradigm away from short-term strain matching toward the induction of cross-reactive neutralizing antibodies, broadly reactive CD4^+^ and CD8^+^ T cell responses, and durable immune memory capable of buffering against future viral evolution and zoonotic spillover.^236^^,^^240^

To achieve this goal, broadly protective vaccine strategies emphasize antigen breadth and immune focus rather than reliance on a single dominant antigen. Key approaches include consensus sequence design and computational antigen discovery, which leverage bioinformatics and machine learning to identify antigen sequences that maximize coverage across viral diversity; mosaic antigen strategies that present multiple variant-derived antigens simultaneously to enhance cross-reactive B cell priming; and nanoparticle-based multivalent display systems that increase antigen valency and promote affinity maturation.^236^^,^^241^, ^242^, ^243^ In addition, increasing attention has been directed toward incorporating conserved non-spike antigens, such as nucleoprotein or non-structural proteins encoded by ORF1ab, to strengthen cross-reactive T cell immunity and complement antibody-mediated protection.^244^^,^^245^ In addition to antigen composition, immune education strategies play a critical role in shaping breadth. Sequential vaccination approaches, in which the immune system is exposed to a series of related but antigenically distinct immunogens, have emerged as a complementary strategy to guide immune focusing toward conserved epitopes.^246^^,^^247^ Originally explored in universal influenza vaccine development, sequential immunization has recently been applied to coronaviruses, where iterative exposure to divergent spike or RBD variants enhances antibody breadth and maturation in preclinical models. When combined with multivalent antigen presentation, such approaches may further refine immune specificity and durability. Equally important is the choice of antigen delivery platform. Multiple technologies—including protein subunit, viral vector, mRNA, and nanoparticle-based platforms—are currently under investigation.^248^ Among these, mRNA–LNP delivery systems have emerged as particularly promising for pan-coronavirus vaccine development because of their modularity, scalability, and ability to encode complex or multivalent antigens, enabling rapid iterative optimization.^242^

In parallel with universal vaccine approaches, combined or multivalent respiratory virus vaccines represent a pragmatic strategy for broadening protection at the population level. By incorporating antigens from multiple respiratory pathogens into a single formulation, combination vaccines enable simultaneous prevention of several diseases, reduce the need for repeated immunizations, simplify vaccine logistics, and improve vaccination coverage. Such strategies are especially relevant in the context of co-circulating respiratory viruses and seasonal vaccination programs.^249^, ^250^, ^251^

**Figure 3:**
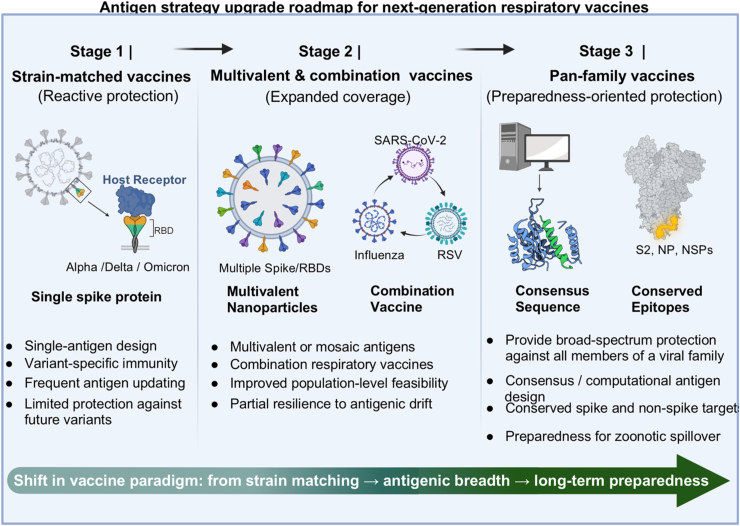
Antigen strategy upgrade roadmap for next-generation respiratory vaccines. Schematic overview of the conceptual evolution in antigen design for respiratory vaccines, transitioning from reactive strain-matched approaches toward preparedness-oriented and broadly protective strategies. Stage 1 represents conventional strain-matched vaccines centered on a single dominant antigen (for example, the spike protein), which confer variant-specific immunity but require iterative antigen updates and provide limited coverage against antigenic drift or future spillover events. Stage 2 illustrates multivalent and combination approaches that incorporate multiple spike or RBD antigens and/or target multiple respiratory pathogens (such as SARS-CoV-2, influenza virus and RSV). These strategies broaden population-level coverage and enhance resilience to antigenic variation, although protection remains partially strain dependent. Stage 3 depicts next-generation preparedness-oriented vaccines that integrate conserved antigens across viral families (including the S2 subunit, nucleoprotein and selected non-structural proteins) and leverage mucosal delivery to establish immunity at the URT. Such approaches emphasize consensus or computationally optimized antigen design to promote SIgA and TRM cells, with the aim of achieving durable protection against both circulating variants and newly emerging zoonotic viruses. The scheme was generated using Biorender.

Collectively, these advances reflect a broader paradigm shift in respiratory vaccine development. Rather than focusing exclusively on updating strain-matched vaccines, next-generation strategies prioritize antigenic breadth, immune durability, and preparedness for future emergence. In this framework, broadly protective and multivalent vaccines—particularly when paired with RM delivery—form the foundation of long-term pandemic preparedness, reducing reliance on repeated development and deployment of narrowly targeted vaccines. This conceptual progression—from strain-matched design to multivalent and preparedness-oriented antigen strategies—is schematically summarized in Figure 3.

## Ensuring the safety of intranasal vaccination for durable and population-wide use

Safety remains a critical determinant of successful intranasal vaccine translation. Although many intranasal vaccines have demonstrated acceptable safety profiles in preclinical and clinical studies, the anatomical proximity of the nasal cavity to the central nervous system (CNS) raises concerns about potential nose-to-brain transport.^149^^,^^252^^,^^253^ Intranasally administered antigens or adjuvants may reach the olfactory bulb and CNS through the olfactory epithelium or adjacent tissues, potentially leading to neurological adverse effects.^252^^,^^254^ For instance, post-marketing surveillance of an intranasal influenza vaccine formulated with a detoxified derivative of the heat-labile enterotoxin (LT) reported cases of facial nerve paralysis, which ultimately led to its withdrawal from the market and highlighted the potential neurotoxicity associated with certain toxin-based adjuvants.^255^^,^^256^

To date, FluMist Quadrivalent remains the only FDA-approved intranasal vaccine, and even this live-attenuated platform is restricted to selected populations because of safety considerations.^257^^,^^258^ In contrast, recombinant protein-based subunit vaccines, which lack intact pathogens, are widely regarded as safer alternatives; however, their relatively low immunogenicity necessitates the use of adjuvants to elicit strong and durable immune responses. Therefore, the development of safe and effective intranasal adjuvants has become key to improving the efficacy of subunit vaccines.^1^ Currently, adjuvant development strategies primarily include utilizing adjuvants already approved for parenteral vaccines (such as MF59 and CpG 1018), as well as developing novel mucosal adjuvants based on PRR agonists, polymers, or microbial sources.^1^^,^^259^, ^260^, ^261^, ^262^ In parallel, nanoparticle- and carrier-based delivery systems have been engineered to enhance nasal mucosal penetration or prolong antigen residence time, thereby improving vaccine efficacy. Nevertheless, these strategies may also increase the likelihood of transport through the nose-to-brain pathway. Therefore, rigorous evaluation of particle size, surface charge, composition, and long-term accumulation is essential to prevent local toxicity and potential long-term adverse effects.^263^ Overall, before entering clinical trials, candidate intranasal vaccines must undergo comprehensive safety assessments to ensure that neither antigens nor adjuvants adversely affect the CNS.

Beyond CNS-related concerns, additional safety factors should be considered for intranasal vaccines, including interindividual variability in nasal deposition, potential entry of vaccine components into the LRT, or post-vaccination shedding (particularly pronounced with attenuated live vaccine platforms).^264^ Variations in nasal anatomy, breathing patterns, and delivery devices can significantly influence antigen distribution within the upper and lower airways, thereby affecting both vaccine efficacy and tolerability.^265^^,^^266^ These considerations are especially critical for individuals with underlying airway hyperresponsiveness, for whom intranasal vaccination has historically warranted cautious evaluation.^267^

## Outlook

Recent advances in vaccine platform technologies have reshaped the landscape of respiratory pathogen prevention, and recurrent outbreaks of pathogens such as SARS-CoV-2 and influenza have further clarified the pivotal role of the RM immune system in blocking infection and transmission. However, despite promising preclinical data, the clinical translation of many intranasal and mucosal vaccine candidates remains challenging, underscoring a persistent gap between conceptual promise and clinical realization.^221^^,^^224^^,^^268^^,^^269^ For example, intranasal ChAdOx1 nCoV-19 conferred strong protection in preclinical challenge models, with reduced viral shedding and transmission in hamsters and macaques, yet in a Phase 1 trial, it was well tolerated but induced only modest and inconsistent mucosal and systemic antibody responses in most participants, highlighting a clear discrepancy between animal efficacy and human immunogenicity.^270^^,^^271^ Similarly, the intranasal live-attenuated influenza vector-based SARS-CoV-2 vaccine dNS1-RBD (Pneucolin®) demonstrated strong immunogenicity and protection in animal studies but achieved only modest efficacy (∼28%; 95% CI 3.4–46.6) against symptomatic infection in a multicentre Phase 3 trial in adults, despite an acceptable safety profile.^272^^,^^273^

A major barrier lies in the low efficiency and pronounced heterogeneity of antigen delivery at mucosal surfaces.^220^ Unlike intramuscular injection, where antigen dose and tissue exposure are relatively well controlled, RM delivery is profoundly influenced by airway anatomy, airflow dynamics, mucus composition, and rapid mucociliary clearance, resulting in substantial inter-individual variability in antigen deposition and residence time.^220^^,^^274^^,^^275^ Consequently, only a fraction of the administered antigen may reach inductive sites, limiting sustained activation of APCs and the consistency of downstream immune responses.^224^^,^^275^ In parallel, the RM immune system is intrinsically shaped toward tolerance, reflecting its constant exposure to environmental antigens and commensal microorganisms. This mucosal tolerogenic bias constrains both the magnitude and durability of vaccine-induced immunity, particularly in the absence of sufficient innate immune stimulation. As a result, although RM vaccines can induce secretory IgA and TRM cells, these responses are frequently transient, and efforts to enhance durability through increased adjuvanticity raise concerns regarding local reactogenicity, epithelial integrity, and long-term safety.^269^^,^^275^

Beyond these biological constraints, the lack of well-defined correlates of mucosal protection remains another major translational barrier. Although secretory IgA and TRM cells are widely regarded as key mediators of RM immunity, no standardized or universally accepted mucosal correlates of protection have yet been established. Persistent heterogeneity in sampling sites, collection methods, and analytical platforms continues to limit cross-study comparability and hampers the identification of robust, predictive biomarkers.^221^^,^^276^ In addition, long-term longitudinal studies of RM vaccine-induced immune memory remain scarce. While some evidence suggests that mucosal vaccination can generate durable immune memory, the mechanisms governing the maintenance of such responses—or the conditions under which short-lived immunity can be converted into long-term protection—remain poorly understood.^34^^,^^277^ Translational complexity is further amplified by the poor predictive validity of conventional preclinical animal models, including mice, ferrets, and non-human primates, that only recapitulate discrete components of human airway anatomy, airflow dynamics, and immune organization.^221^ Such limitations often result in an overestimation of therapeutic efficacy and durability when extrapolating preclinical outcomes to human clinical trials.

Looking forward, overcoming these barriers will require integrated, technology-driven strategies. First, rational RM vaccine design should be guided by high-resolution maps of the human RM immune system. Single-cell sequencing and spatial transcriptomics offer unprecedented opportunities to construct comprehensive mucosal immune atlases, defining the spatial distribution and functional states of epithelial cells, APCs, and lymphoid niches across distinct airway compartments.^278^ Such atlases could enable vaccine designs that deliberately target specific immune cell populations, including M cells and defined dendritic cell subsets, rather than relying on empirical formulation approaches.^73^^,^^279^ Second, innovative delivery strategies beyond conventional nasal sprays or liquid aerosols warrant systematic investigation. Alternative platforms such as dry powder inhalers may improve antigen stability, prolong mucosal residence time, and enhance distribution uniformity throughout the respiratory tract, while reducing cold-chain dependence.^280^ Third, optimization of immunization strategies, particularly combined systemic–mucosal regimens (for example, intramuscular priming followed by intranasal boosting), holds promise for recruiting pre-existing systemic immune memory into mucosal sites, potentially enabling more durable local immunity and more effective interruption of transmission.^199^^,^^200^ Finally, progress toward clinical implementation will depend on advances in regulatory science, including the establishment of standardized clinical endpoints for RM vaccines.^281^^,^^282^ Harmonized measurements of mucosal IgA responses, feasible surrogates of tissue-resident memory and virological endpoints such as reduced viral shedding, will be essential to accelerate clinical evaluation, facilitate cross-trial comparisons, and support regulatory approval. Together, these efforts may help bridge the gap between conceptual promise and clinical impact, enabling RM vaccines to fulfill their potential in controlling respiratory pathogens.

## CRediT authorship contribution statement

**Yu Zhang:** Writing – original draft. **Zhiruo Song:** Visualization. **Weiqi Hong:** Visualization. **Xuemei He:** Writing – review & editing. **Xiawei Wei:** Writing – review & editing, Funding acquisition.

## Data availability

The datasets used and analyzed during the current study are available from the corresponding author on reasonable request.

## Funding

This work was supported by the 10.13039/501100012166National Key Research and Development Program of China (No. 2024YFC2310700 to X.W.), 1.3.5 project for disciplines of excellence from West China Hospital of Sichuan University (China) (No. ZYGD23038 to X.W), the Project of the 10.13039/501100004829Science and Technology Department of Sichuan Province, China (No. 2023NSFSC1655 to X.H), the 10.13039/501100001809National Natural Science Foundation of China Young Student Basic Research Program (China) (No. 323B2050 to W.H), the 10.13039/501100002858China Postdoctoral Science Foundation (China) (No. 2025M782195 to W.H) and the Sichuan Science and Technology Program (China) (No. 2026NSFSC1763 to W.H).

## Conflict of interests

Dr. Xiawei Wei is a member of the *Genes & Diseases* Editorial Board. To minimize bias, Dr. Xiawei Wei was excluded from all editorial decision-making related to the acceptance of this article for publication. The remaining authors declare no conflict of interests.
